# RNA-seq based T cell repertoire extraction compared with TCR-seq

**DOI:** 10.1093/oxfimm/iqaf001

**Published:** 2025-03-26

**Authors:** Linoy Menda Dabran, Alona Zilberberg, Sol Efroni

**Affiliations:** The Mina & Everard Goodman Faculty of Life Sciences, Bar-Ilan University, Ramat-Gan 5290002, Israel; The Mina & Everard Goodman Faculty of Life Sciences, Bar-Ilan University, Ramat-Gan 5290002, Israel; The Mina & Everard Goodman Faculty of Life Sciences, Bar-Ilan University, Ramat-Gan 5290002, Israel

**Keywords:** RNA-seq, TCR-seq, T cell repertoire extraction, AIRR-seq

## Abstract

The purpose of this study is to evaluate the feasibility of using RNA sequencing data as substrate for the computational extraction of T cell receptor sequences. Data from hundreds of thousands of samples is available as RNA sequencing. However, the use of these data for repertoires has not been contrasted against a gold standard. We conducted a benchmarking analysis, comparing T cell receptor data extracted from RNA sequencing to those obtained from T cell receptor sequencing (as gold standard) of the same tissue samples. The focus was on the extraction of Complementarity-Determining Region 3 (CDR3) sequences. To evaluate the influence of sequencing read lengths, samples were analyzed using both 75 base pair single-end and 150 base pair paired-end sequencing methods. In addition we calculated T cell abundance in these samples to test for any correlation between reads and abundance. The findings reveal a significant, perhaps too great, discrepancy between the ability to extract Complementarity-Determining Region 3 sequences from RNA sequencing data and the results obtained from TCR sequencing. The lack of significant improvement with longer read lengths, combined with the absence of correlation to T cell abundance, emphasize the necessity of using T cell receptor sequencing methodologies.

## Introduction

Over the past two decades, hundreds of thousands [1] of samples have been analyzed for the RNA content using RNA-Sequencing (RNA-Seq) [2]. The field of AIRR-Seq, hungry for data to demonstrate the utility [3] of TCR and BCR data, and to develop second order tools to learn from repertoires, has produced clever tools to mine RNA-seq data, for the possible available receptor sequences contained in these non-targeted assays. This, in contrast with targeted sequencing (e.g. TCR-Seq [4]) where repertoire sequences are abundant. Multiple tools are now available for the task of extracting TCR sequences out of RNA-seq reads [5].

RNA-seq is designed to sequence all transcripts, without any enrichment of TCR sequences. As such, it may not capture full lengths of the VDJ, nor would it capture most of the sequences. Tools such as (the specific implementation within) MiXCR [6], and TRUST4 [7] are most often used for this task. These tools provide identification and annotation of V, D, and J gene segments, as well as the determination of the CDR3 region. As this work is not aimed at a specific tool, but rather at benchmarking the concept of RNA-receptor-extraction, we did not try all the tools included in [5] but rather used MiXCR and TRUST4 as representative of what is possible. These tools are the most extensively used tools.

TRUST4 specializes in analyzing TCR and BCR sequences from unselected RNA sequencing data across various tissues, including tumors. It focuses on *de novo* assembly of key genes like V, J, and C genes, leading to CDR3, to generate consensus contigs. MiXCR uses a different operational approach and creates clonotypes based on diverse criteria, employs alignment algorithms, adjusts to library architectures, and reconstructs CDR3 regions.

The gold standard for discovering TCR sequences within any tissue is TCR-seq. This is achieved through amplification of cDNA or genomic DNA from the TCR β and α chains using pre-designed primers, followed by sequencing [4, 8].

To learn about the differences between RNA-seq extracted TCRs and TCR-Seq extracted TCRS, we produced these datasets:

1. RNA-seq of 19 samples from ovarian cancer tumors. Sequencing was performed using single-end RNA-seq. Reads were 75 bp long.
2. RNA-seq of four samples from the set detailed in (1), This time, the samples were sequenced using paired-end 150 bp long reads.
3. TCR-seq of the same set of four samples detailed in (2).

The aim of this study is to observe the differences between the two RNA-seq based computational methods, and the TCR-seq gold standard. The minimal nature of the output from RNA-seq (a very small number of TCRs is usually obtained, as well as in this case), lead us to focus solely on the CDR3 region, and the ability of the different tools to identify it, regardless of any differences in V, J. The reason we used two different lengths for reads in RNA-seq was to see if by increasing the capture probability of V, J regions would increase the yield of CDR3 outcomes. To this end, we used paired end sequencing and increased sequencing depth with the 150PE configuration.

## Methods

### RNA purification

#### Sample preparation

Total RNA was extracted from 19 frozen ovarian cancer biopsy samples. The samples were first thawed on ice and homogenized using a tissue homogenizer in the presence of Trizol (Invitrogen). The homogenized samples underwent an RNA purification procedure followed by DNase digestion to eliminate any genomic DNA contamination. RNA was eluted in RNase-free water.

#### Quality and quantity assessment

RNA concentration was measured using a Qubit RNA Assay Kit (Thermo Fisher Scientific), and RNA integrity was assessed using a tapestation with RNA Nano Chips (Agilent Technologies). Samples were evaluated for their RNA Integrity Number (RIN) values and were used for subsequent library preparation.

### Library preparation for RNA sequencing

Libraries were prepared using the SMARTer^®^ Stranded Total RNA Sample Prep Kit—HI Mammalian (Takara Bio USA) according to the manufacturer’s instructions. 1 µg of total RNA from each sample was used as the input for library preparation. First-strand cDNA synthesis was performed using random priming and SMART (Switching Mechanism at 5' end of RNA Template) technology to ensure efficient synthesis and strand-specificity. The cDNA was amplified using PCR with the kit-supplied primers, followed by purification of the PCR products using AMPure XP beads (Beckman Coulter). The cDNA was fragmented to the desired size using the fragmentation reagent provided in the kit. Sequencing adapters were then ligated to the fragmented cDNA. The second strand of cDNA was synthesized, incorporating dUTP for strand specificity. The resulting double-stranded cDNA was purified using AMPure XP beads. A final round of PCR amplification was performed to enrich adapter-ligated fragments, followed by purification using AMPure XP beads. The quality and quantity of the final libraries were assessed using the tapestation with High Sensitivity DNA Chips and the Qubit dsDNA HS Assay Kit.

### Sequencing

#### Nextseq 500 sequencing

##### 75 bp Single-End sequencing

Libraries were diluted to the appropriate concentration for the Nextseq 500 sequencing platform. The libraries were loaded onto the Nextseq 500 and sequenced in a 75 bp single-end format, targeting 20,000 reads per sample.

##### 150 bp Paired-End sequencing

Libraries were similarly diluted and loaded onto the Illumina sequencer using the 300-cycle Nextseq 500/550 Mid Output Kit v2.5 with pair-end, 2 × 150 base pair reads targeting 40,000 reads per sample.

### Library preparation for TCR sequencing

A fixed total RNA concentration of 200 ng from each sample was subjected to the SMARTer Human TCR a/b Profiling Kit V2 (Takara Bio). This kit enables the analysis of TCR repertoires from bulk RNA samples. It generates Illumina compatible sequencing libraries. The TCR sequencing library is then size-selected and purified using AMPure XP beads. The generated libraries were measured for their DNA concentration by qubit and assessed for their sizes using Tapestation. This allows to pool together 24 libraries on each single flow cell, preserving an equal representation of each single library at the final pool.

#### 150 bp Paired-end sequencing

Sequencing was performed on an Illumina sequencer using the 300-cycle Nextseq 500/550 Mid Output Kit v2.5 with pair-end, 2 × 150 base pair reads. That specific format enables us to capture the CDR3 domain of each TCR α/β transcript.

### RNA-seq data processing

#### Salmon and Ensembl

During preprocessing, to produce expression data, we used the Salmon [9] tool (“salmon_version”: “1.10.2”), which quantifies transcript expression from RNA-seq data. Subsequently, we processed the outputs through a custom script, merging results from each file and storing gene IDs along with their corresponding Transcripts Per Million (TPM) values obtained from Salmon. For accurate gene identification, the script mapped gene IDs to their corresponding names using a MART file downloaded from Ensembl [10], which served as the key-value reference for human genes (GRCh38.p14).

#### xCell

Transcript expression data from RNA-seq was analyzed using the xCell [11] webtool to perform cell type enrichment analysis and infer potential cell subsets based on gene signatures. After obtaining the xCell outputs, only T cell types were counted to calculate the T cell enrichment for each sample.

#### RNA-Seq-based repertoire profiling tools

TCRs were extracted from RNA-Seq data using MiXCR (v4.5.0) and TRUST4 (v1.0.13).

## Results

**Figure 1.**
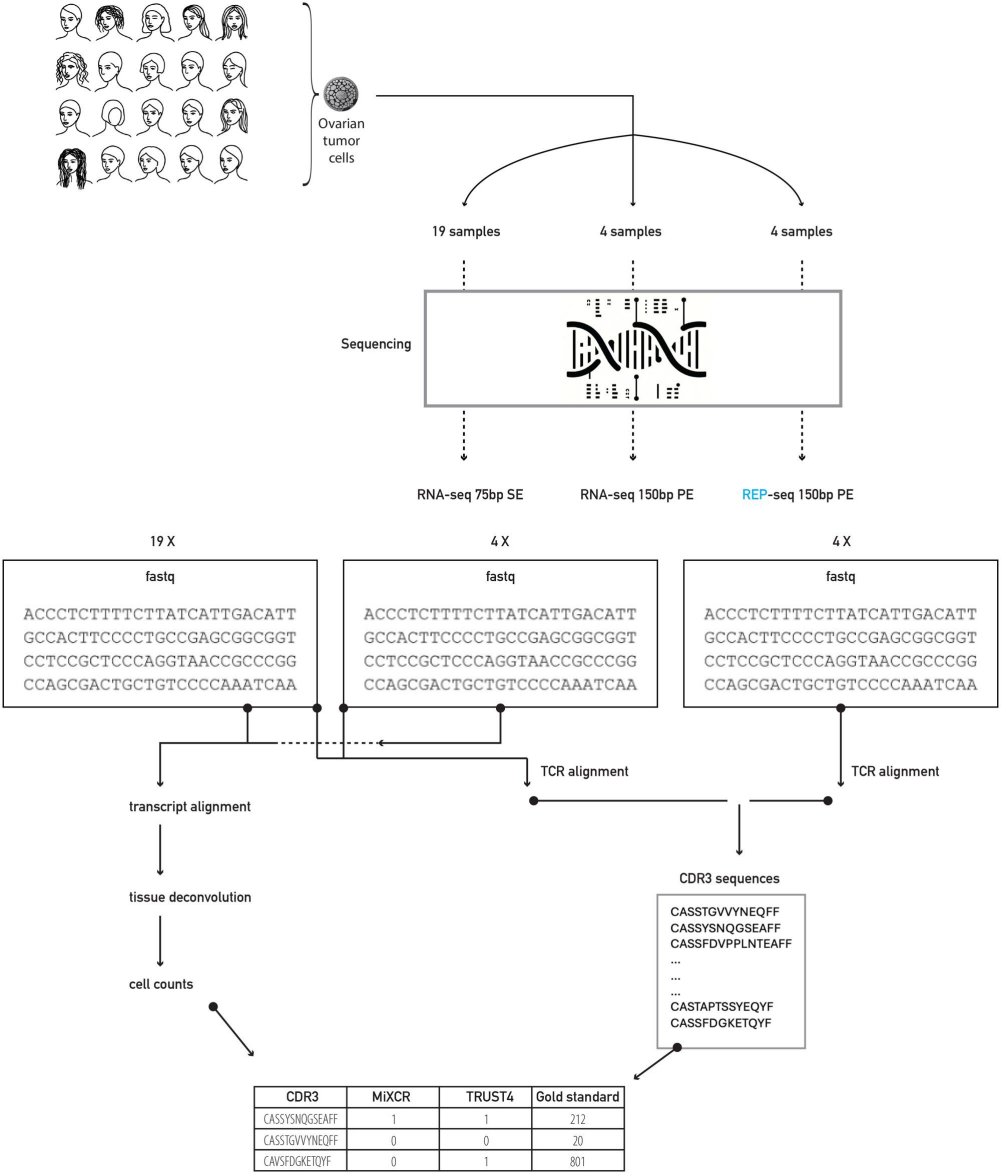
Workflow Overview. The figure illustrates the workflow involving three datasets. Dataset 1 include RNA-seq single-end reads (75 bp) containing 19 samples. Dataset 2 includes 4 samples from Dataset 1 that were sequenced with RNA-seq paired-end reads (150 bp). Dataset 3 consists of the same 4 samples sequenced with TCR-seq data. For extracting CDR3 sequences, Datasets 1 and 2 were processed using MiXCR and TRUST4, whereas Dataset 3, processed using MiXCR, served as the gold standard for CDR3 sequence extraction. To determine T-cell richness, Datasets 1 and 2 were aligned and analyzed using xCell, following transcript quantification with salmon.

**Figure 2.**
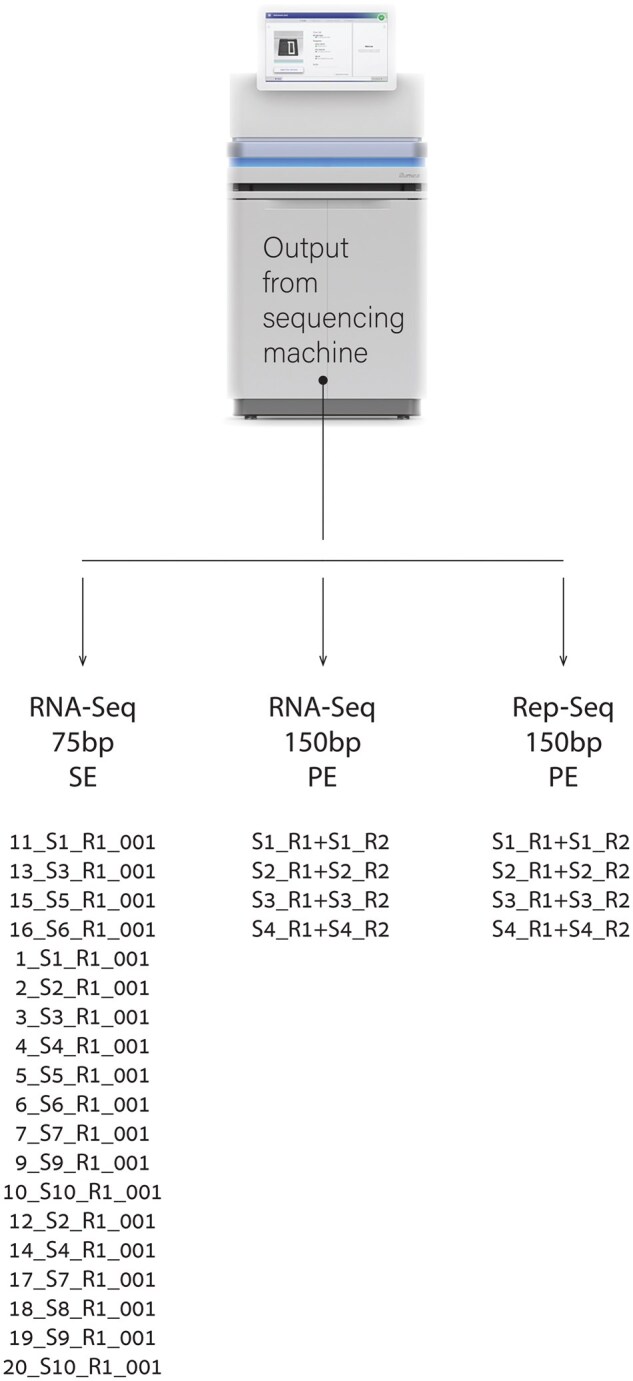
An overview of the dataset, involving various sequencing approaches applied to ovarian tumor tissue. The dataset includes 19 samples which were RNA-sequenced with a read length of 75 base pairs and 4 samples were RNA-sequenced with a read length of 150 bp as well as TCR-Seq’ed with a 150 bp read length.

We used ovarian tumor tissue we collected through collaboration with the BioBank of Sheba medical center. Our analysis focuses on the TCR β-chain repertoire. Specifically, we compare output from the different tools, used to analyze the repertoires of 19 samples (Figs 1 and 2). The samples were sequenced using three different approaches: (i) all samples were sequenced using single-end 75 bp RNA-seq. In addition, (ii) samples 11, 13, 15, and 16 were randomly selected and sequenced using paired-end 150 bp RNA-seq, as well as (iii) TCR-seq at 150 bp resolution.

**Figure 3.**
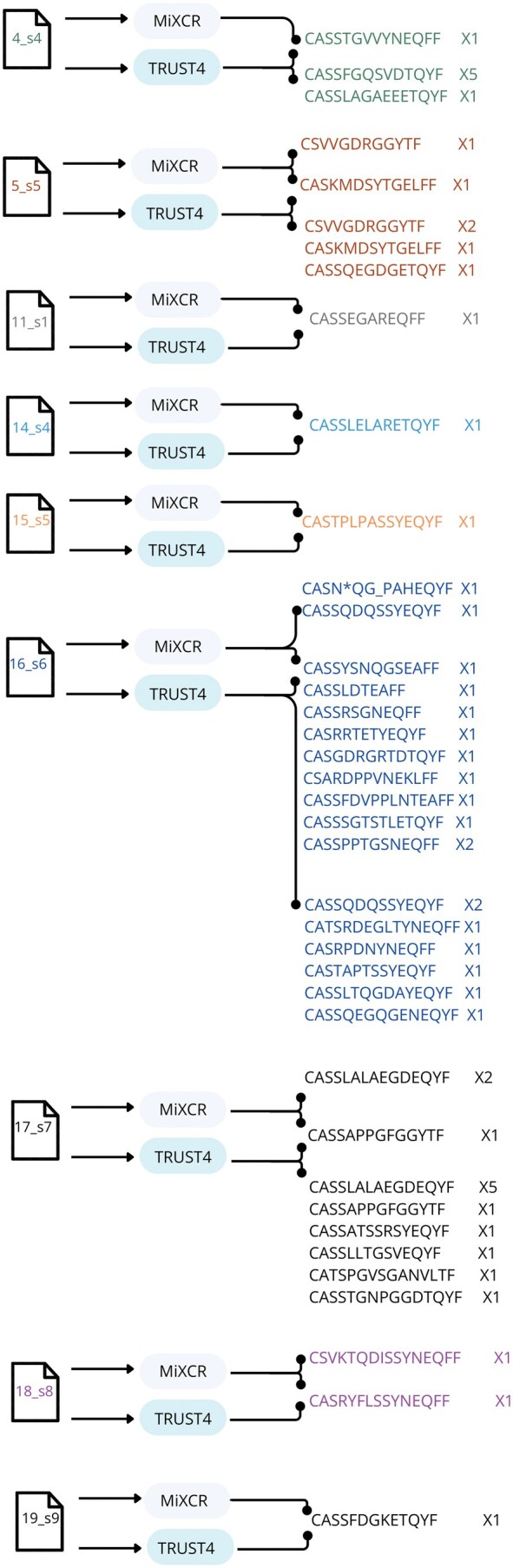
CDR3 sequences and the number of reads extracted from the 75 bp RNA-seq, compared using MIXCR and Trust4 tools.

We started by analyzing the 19 (75 bp single-end) samples, for their CDR3 sequences. The results (Fig. 3), include output from MiXCR and from TRUST4. Samples not listed in the figure were omitted due to zero extracted CDR3 sequences.

The rearranged TCR β chain is approximately 500 bases in length [12]. Since sequencing reads generally are shorter (with exceptions) than this segment, there is influence of read length over the reconstructed TCR-seq output. We are interested in exploring the potential read length effect on TCR data from RNA-seq experimentation.

**Figure 4.**
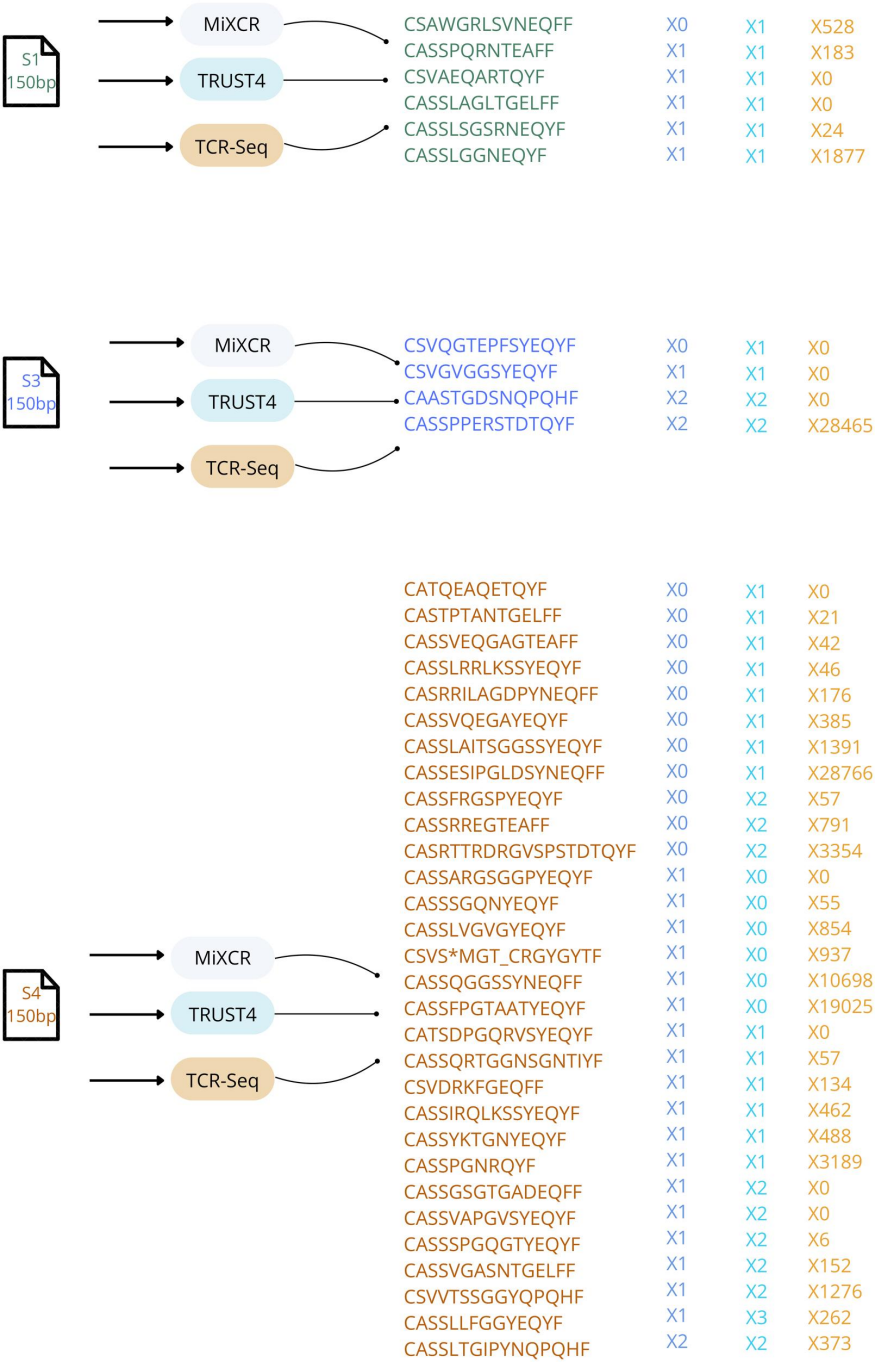
CDR3 sequences and the number of reads extracted from the 150 bp RNA-seq and TCR-seq datasets, compared with outputs from MiXCR and TRUST4. Only CDR3 sequences found in MiXCR or Trust4 RNA-seq were included in the figure.

Figure 4 presents output from samples that were sequenced using paired-end 150 bp RNA-seq and samples studied using TCR-Seq. For TCR-Seq, post sequencing analysis has been performed using MiXCR (using the TAKARA BIO flag). The figure only includes CDR3 sequences that were identified in the RNA-Seq analyses (either by MIXCR or by TRUST4). Sequences that were exclusively identified by TCR-seq and not re-encountered in the RNA-seq are omitted from the figure simply to provide a clear representation. As previously noted, samples with zero extracted CDR3 sequences excluded from the figure. For example, sample S2 is not included since neither MiXCR nor TRUST4 found any CDR3 sequences in its RNA-Seq.

Table 1 provides unfiltered sequence abundance over the various samples. This is to allow for unbiased comparison between the number of sequences obtained through the different read-lengths used.

**Table 1.** Number of unique CDR3 sequences per sample per technology.

Sample	Sequencing Technique	Unique CDR3 count from MIXCR	Unique CDR3 count from TRUST4
11_S1_R1_001	RNA-seq 75 bp	1	1
S1	RNA-seq 150 bp	5	6
S1	REP-seq	2795	–
13_S3_R1_001	RNA-seq 75 bp	0	0
S2	RNA-seq 150 bp	0	0
S2	REP-seq	471	–
15_S5_R1_001	RNA-seq 75 bp	1	1
S3	RNA-seq 150 bp	3	4
S3	REP-seq	1257	–
16_S6_R1_001	RNA-seq 75 bp	11	15
S4	RNA-seq 150 bp	27	24
S4	REP-seq	39499	–
1_S1_R1_001	RNA-seq 75 bp	0	0
2_S2_R1_001	RNA-seq 75 bp	0	0
3_S3_R1_001	RNA-seq 75 bp	0	0
4_S4_R1_001	RNA-seq 75 bp	1	3
5_S5_R1_001	RNA-seq 75 bp	2	3
6_S6_R1_001	RNA-seq 75 bp	0	0
7_S7_R1_001	RNA-seq 75 bp	0	0
9_S9_R1_001	RNA-seq 75 bp	0	0
10_S1_R1_001	RNA-seq 75 bp	0	0
12_S2_R1_001	RNA-seq 75 bp	0	0
14_S4_R1_001	RNA-seq 75 bp	1	1
17_S7_R1_001	RNA-seq 75 bp	2	6
18_S8_R1_001	RNA-seq 75 bp	2	1
19_S9_R1_001	RNA-seq 75 bp	1	1
20_S10_R1_001	RNA-seq 75 bp	0	0

Our analysis aims to explore associations between the richness of the repertoire, determined by TCR-seq per sample, and the numbers of CDR3 sequences obtained by the RNA-seq TCR extraction tools. To see how the actual tissue composition of the samples is associated with these TCR findings, we used the tool xCell.

xCell has been shown to excel in cell type enrichment analysis, enabling the quantification of the relative abundance of cell types within tissue (see Supplementary Tables 1 and 2). xCell takes a gene expression data file as input and provides enrichment as output.

**Figure 5.**
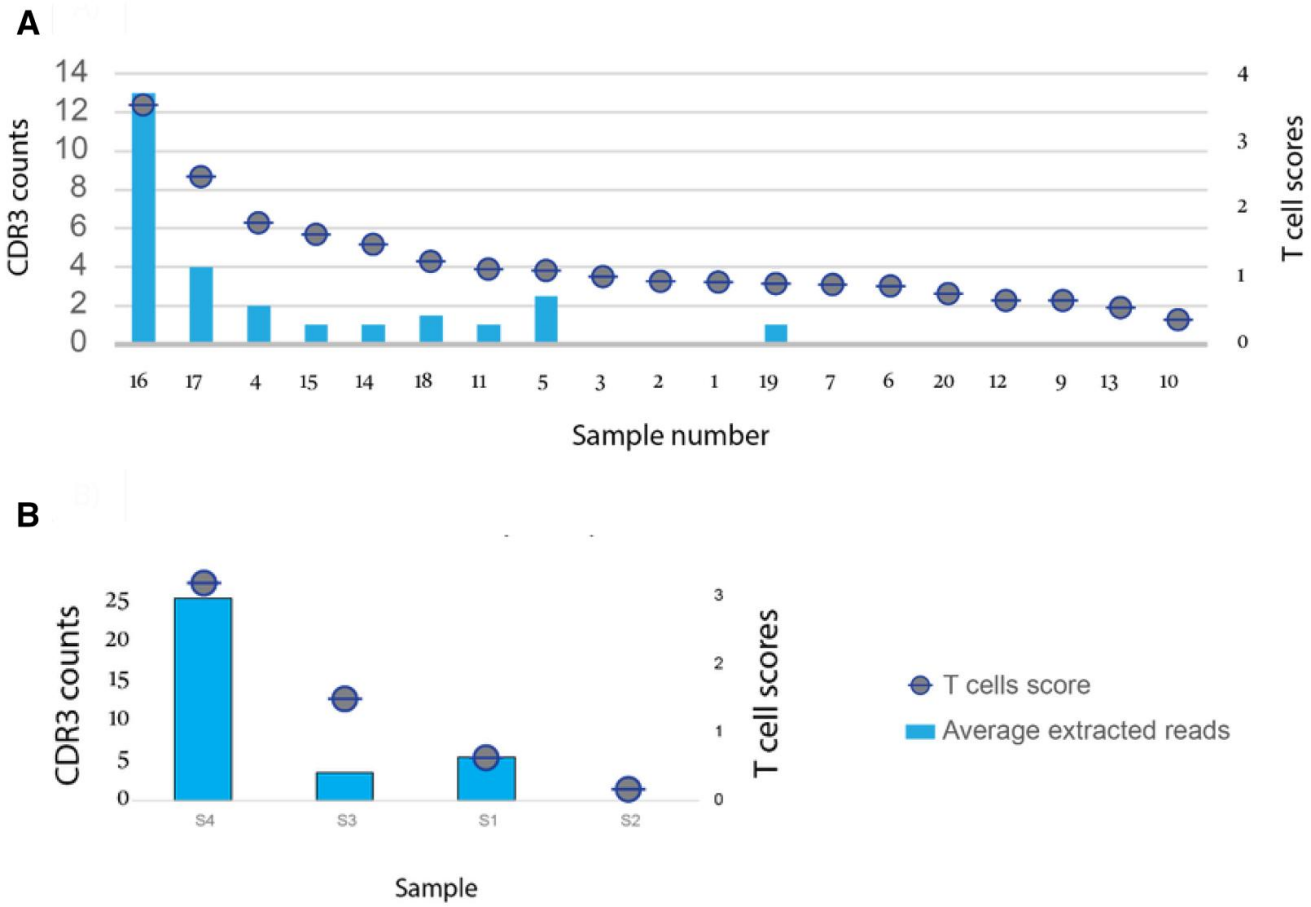
The graphs illustrate the association between T cell scores and CDR3 counts obtained from MiXCR and TRUST4, for (A) SE-75bp samples and for (B) PE-150 bp samples.

To see if the extremely low numbers of extractable CDR3 sequences in RNA-seq were associated with the xCell-explained number of T cells in tissue, we present them side by side in Fig. 5A. In the 75 bp samples, notably, samples 11, 14, and 15 show higher xCell T cell score compared with samples 5 and 19. However, 5 and 19 provided equivalent or greater numbers of CDR3 sequences. Further, while sample 18 gives an xCell T score higher than that for sample 5, the latter produces a larger number of CDR3 sequences. In the 150 bp sample (Fig. 5B) S3, it produces greater T score compared with S1, however, S1 still provides a larger number of CDR3 sequences.

## Discussion

This study aims to evaluate the success rates of TCR CDR3 extraction out of RNA-seq data. Higher rates of success would validate RNA-seq datasets amenable for TCR mining. Given the abundance of RNA-seq datasets, such validation could bring forth much needed samples to the field of AIRR-seq based clinical data mining. However, the findings in our work indicate a significant gap, perhaps too great, between the ability to extract CDR3 sequences from RNA-seq data and their comparison with (gold standard) TCR-seq data. Results show that capturing CDR3 sequences from RNA-seq data using MiXCR or TRUST4, the most used tools, did not yield any significant numbers of CDR3 sequences, in contrast with TCR-seq of the same tissue providing (tens of) thousands of CDR3 sequences. The suboptimal performance of MiXCR in extracting CDR3 sequences from RNA-seq libraries, particularly from tumor biopsy samples, is expected given the inherent limitations of such tissues: T cells typically constitute only about 10% of the tumor microenvironment, and among the ∼20,000 coding genes, TCR alpha and TCR beta transcripts represent a minuscule fraction. This low abundance creates a challenge for RNA-seq approaches, as the competition for sequencing reads among all expressed genes significantly reduces the representation of TCR transcripts. To address this, one could increase tissue input or prioritize TCR sequences. However, tumor biopsies are weight-limited, constraining the feasibility of enriching tissue mass. A more effective solution lies in the application of **Rep-seq** methodologies, which leverage targeted PCR strategies to selectively amplify TCR alpha and beta transcripts from limited biopsy material. This targeted approach significantly enhances the detection and quantification of TCR repertoires, bypassing the limitations of RNA-seq's untargeted nature. We also checked to see the influence of read lengths in this context, and added (to the 75 bp), a set of experiments using 150 bp paired end sequencing with increased depth. However, no significant improvement has been recorded in the ability to extract CDR3 sequences. We also showed that the small amounts of aligned sequences could not be attributed to the numbers of T cells. This has been shown by calculating (using xCell deconvolution) the abundance of T-cells within a given sample and comparing these scores with the number of extracted CDR3 sequences. These findings suggest that factors other than T cell abundance may cause the limitations in the efficiency of capturing CDR3 sequences. In conclusion, the utility of RNA-seq is very limited within the context of learning TCRs studies, yielding only a small number of CDR3s, in a sample where those are abundant. The minimal number of TCRs obtained and the lack of significant improvement with longer read lengths, together with the lack of correlation to T-cell abundance, highlight the need for TCR-seq methodologies when aiming at TCR-based conclusions.

## Supplementary Material

iqaf001_Supplementary_Data

## Data Availability

The data used will be shared upon request. The dataset supporting the results of this article is available at GigaDB https://github.com/LinoyMenda/Benchmarking_RNASeq/tree/main. Ovarian Cancer RNA-seq 150 bp (samples S1, S2, S3) - Zenodo id 11479616 (currently private). Ovarian Cancer RNA-seq 150 bp (sample S4) & REP-seq—Zenodo id 11480282 (currently private). Ovarian Cancer RNA-seq 75 bp (samples S1, S2, S3, S4, S5, S6) - Zenodo id 11495709 (currently private). Ovarian Cancer RNA-seq 75 bp (samples S7, S9, S10, S11, S12, S13) - Zenodo id 11495987 (currently private). Ovarian Cancer RNA-seq 75 bp (samples S14, S15, S16, S17, S18, S19, S20) - Zenodo id 11496319 (currently private).
