# Supplementary material for: RNA-seq based T cell repertoire extraction compared with TCR-seq
Source: Oxf Open Immunol. 2025 Mar 26;6(1):iqaf001. doi: 10.1093/oxfimm/iqaf001 (PMC11972113; doi:10.1093/oxfimm/iqaf001)
Supplement: iqaf001_Supplementary_Data [file iqaf001_supplementary_data.zip › Supplementary data.docx]

**Supplementary data**

**Table 1 – xCell output for 75 bp sequencing samples**

X10 X11 X12 X13 X14 X15 X16 X17 X18 X19 X1 X20 X2 X3 X4 X5 X6 X7 X8 X9

Adipocytes 0.2132 0.2023 0.0438 0.0532 0.6002 0.1249 0.045 0.4074 0.1923 0.0491 0.1088 0.0874 0.0593 0.0569 0.0435 0 0.0614 0.1461 0.1247 0

Astrocytes 0.0524 0.0349 0.0478 0 0 0 0.0314 0.003 0.0499 0.1024 0.1008 0.2363 0 0.1183 0 0.0971 0.0782 0.0044 0.0275 0

B-cells 0 0 0.0065 0 0.0117 0.0826 0.1276 0.2175 0.0147 0.0081 0.0453 0.0127 0 0 0.0482 0.0132 0.0413 0 0 0

Basophils 0.2473 0.1891 0.0658 0.2538 0.0564 0.0626 0.3806 0.2453 0.1758 0.092 0 0.0906 0.1117 0.0189 0.1703 0.1458 0.1213 0.2572 0.1592 0.3314

CD4+ T-cells 0.1227 0.122 0 0.0697 0.1872 0.2577 0.5155 0.3333 0.1851 0.1162 0.0753 0.0339 0.1136 0.0783 0.1492 0.0539 0.1256 0.0978 0.1136 0

CD4+ Tcm 0 0 0 0.0523 0.0345 0.1962 0.2034 0.125 0 0 0 0 0 0 0.0788 0 0.0101 0.0152 0 0

CD4+ Tem 0.055 0.0887 0.0724 0 0.3077 0.4044 0.4717 0.3656 0.1677 0.0654 0.0448 0 0 0.106 0.2409 0 0 0.038 0.0591 0

CD4+ memory T-cells 0 0.2438 0.0057 0.0971 0.2987 0.3774 0.7283 0.5363 0.2569 0.2018 0.1937 0.1057 0.1101 0.1819 0.3433 0.2068 0.0966 0.1899 0.1825 0.0648

CD4+ naive T-cells 0.0468 0 0 0 0.0244 0.0054 0.1562 0.0883 0 0 0 0 0 0 0 0 0.0119 0 0.0184 0

CD8+ T-cells 0 0 0 0.0414 0.0216 0 0.1736 0.1591 0 0 0 0.0162 0.0023 0 0.0482 0 0.0075 0.1148 0 0

CD8+ Tcm 0 0.0752 0 0.0498 0.0184 0.0717 0.2708 0.1572 0.1201 0.0496 0.0389 0 0.0232 0.0495 0.0369 0.065 0 0.1233 0.0052 0.0487

CD8+ Tem 0 0.0778 0.0358 0 0.149 0.1191 0.3761 0.2113 0.0517 0.0227 0.0649 0.065 0.0173 0.1241 0.2584 0.0475 0 0.142 0 0

CD8+ naive T-cells 0.0414 0 0.001 0.0272 0 0.0118 0 0 0 0 0 0.0078 0.0683 0 0 0 0.0922 0.0237 0.0601 0.0285

CLP 0.0339 0.0368 0 0.0374 0.0339 0 0.0313 0.0065 0.0628 0.0729 0.0729 0 0.0463 0.0681 0.0376 0.0867 0.0424 0.0394 0.004 0.0299

CMP 0.0748 0.047 0.0727 0.0891 0.0459 0 0 0 0.1395 0.0374 0.037 0 0.1095 0.0448 0 0.0043 0.1521 0.1303 0.1298 0.0459

Chondrocytes 0.2399 0.2121 0.2104 0.0925 0.0833 0 0 0.0924 0.0116 0 0.0591 0.1951 0 0 0.0398 0.0832 0.0668 0.261 0.2168 0

Class-switched memory B-cells 0 0 0 0 0.1095 0.1123 0.1351 0.2547 0 0.0492 0.0305 0.0253 0 0.0051 0.069 0 0 0 0 0

DC 0.023 0.1306 0 0.0026 0.2484 0.0229 0.2443 0.222 0.184 0.1962 0.1154 0.0647 0.0024 0.1749 0.0678 0.0726 0 0.0522 0.0807 0

Endothelial cells 0.0418 0.1484 0 0 0.3124 0.059 0 0.0999 0.1053 0.0203 0.0437 0.0303 0.0557 0.0957 0.0195 0.017 0.0028 0.1209 0.0678 0

Eosinophils 0.003 0.0034 0.2117 0.1199 0.1145 0.3883 0.0996 0.0496 0.0491 0.0146 0 0 0.0914 0.1548 0.0416 0 0.0828 0.1833 0.1848 0

Epithelial cells 0.2023 0.2577 0.4565 0.4949 0.0011 0.4276 0.2872 0.2202 0 0.4989 0.4671 0.3964 0.2382 0.4282 0.4866 0.4411 0.2881 0.3557 0.2306 0.4633

Erythrocytes 0 0.028 0.0398 0 0 0 0 0 0 0 0.0166 0.0652 0.0389 0 0.0177 0.0411 0.0371 0 0 0.0246

Fibroblasts 0.3579 0.282 0 0 0.1903 0 0 0.1449 0.153 0.0095 0 0.0946 0 0 0 0 0.0773 0.2618 0.2286 0

GMP 0 0.1407 0.0057 0 0.0854 0 0.0301 0.0195 0.1424 0.0205 0.0264 0 0.0291 0.0435 0 0.026 0 0.0665 0 0.0021

HSC 0.345 0.3046 0 0.0535 0.5627 0.0023 0 0.1113 0.3878 0 0.0194 0.2174 0.1037 0.0169 0.0049 0 0.0073 0.314 0.0954 0.1656

Hepatocytes 0.0843 0.0037 0.0289 0.0287 0.0098 0.0462 0 0 0.0254 0.0292 0.0268 0 0.0228 0 0.0303 0 0.0169 0.0328 0.0515 0

Keratinocytes 0.0781 0.1163 0.2277 0.2562 0 0.2134 0.1321 0.0666 0 0.2587 0.3185 0.3069 0.1089 0.2202 0.2562 0.198 0.1109 0.2714 0.0875 0.1965

MEP 0 0 0 0 0 0 0 0 0 0 0 0.0056 0.0513 0 0 0.0308 0.0412 0 0 0.1708

MPP 0 0.0066 0.0559 0 0.2184 0.1968 0.3396 0.3158 0.1591 0.1202 0.1406 0 0.1411 0.1235 0.1245 0.0291 0.0128 0 0.0104 0

MSC 0.4309 0.3959 0.0968 0.1638 0.3049 0 0.0952 0.404 0.6449 0.1599 0.3571 0.5051 0.1577 0.5784 0.1325 0.1622 0.1565 0.1882 0.3057 0.3101

Macrophages 0 0.1567 0.0562 0.0468 0.1648 0.037 0.3385 0.1833 0.3471 0.1592 0.2437 0.0962 0.0649 0.1642 0.2304 0.1889 0 0.0499 0.0961 0.0623

Macrophages M1 0 0.0717 0 0 0.0732 0.0158 0.1357 0.0888 0.05 0.0658 0.0645 0.0238 0 0.0392 0.0586 0.0535 0 0.0057 0.005 0.0215

Macrophages M2 0.0478 0.0994 0.1166 0.0944 0.2525 0.1091 0.2529 0.1047 0.4796 0.083 0.1971 0.0725 0.1855 0.0914 0.2037 0.1837 0.1163 0 0.1204 0.0757

Mast cells 0.0281 0 0.0298 0.0207 0.0345 0.0213 0.0331 0.0104 0.0217 0.0073 0.002 0.0054 0.024 0.0061 0.0036 0.0044 0.0248 0.0549 0.029 0.0039

Megakaryocytes 0.1965 0.0373 0.0567 0.056 0.1232 0.0695 0 0 0 0.0292 0 0 0.1011 0.0519 0 0.0162 0.0601 0.0038 0.0368 0.0409

Melanocytes 0.1108 0.0697 0.0159 0.0565 0.0678 0.0276 0.0466 0.0567 0.0691 0.0751 0.0938 0.0395 0.0264 0.014 0.0714 0.0631 0.0435 0.0827 0.0619 0

Memory B-cells 0 0 0 0 0 0.0101 0.0103 0.1004 0 0 0 0.047 0 0 0 0 0 0 0 0

Mesangial cells 5e-04 0.1065 0.1018 0.1581 0.021 0 0.09 0.0809 0 0.0951 0.1003 0 0.0324 0.1649 0.1378 0.1132 0.0585 0.0556 0 0.0937

Monocytes 0.0285 0.1489 0 0.0278 0.2299 0.1591 0.0503 0.0841 0.1769 0.0773 0.0437 0.0062 0 0.0949 0.1203 0 0 0 0.0478 0

Myocytes 0.0753 0.0598 0.0644 0.0183 0.0358 0 0 0 0.0516 0 0.041 0.0607 0.1346 0 7e-04 0.0205 0.0885 0.0181 0.0154 0.0196

NK cells 0 0 0 0 0 0.0397 0.1013 0 0.0277 0 0 0 0.0028 0 0.1161 0.0428 0.0213 0 0 0.0916

NKT 0 0 0.1001 0.0149 0.2665 0.0068 0.044 0.0693 0.1073 0 0 0.0658 0.0642 0.0865 0.0708 0.2321 0.0395 0.0053 0.0639 0.025

Neurons 0.0565 0.0301 0.0531 0.0463 0.0272 0.0062 0 0.0227 0.0422 0.0258 0.0174 0.0599 0.0304 0 0 0.026 0.0711 0.0495 0.0553 0.0208

Neutrophils 0.0313 0.0565 0 0.0137 0.16 0.4073 0.1052 0.113 0.0429 0.0627 0.0045 0.0436 0.0049 0.0295 0.1203 0.0076 0 0.0735 0.0802 0.054

Osteoblast 0.0067 0 0.2204 0.1478 0.0287 0 0 0 0.0032 0.173 0.0142 0.0753 0.2284 0 0 0.1757 0.1809 0 0.0983 0.3367

Pericytes 0.085 0.0762 0.0466 0.0133 0.0914 0 0 0.0226 0.0661 0.0363 0.0395 0.1334 0.0301 0.1224 0.0435 0 0.0121 0.1481 0.0475 0.0299

Plasma cells 0 0 0.106 0 0 0.1524 0.0705 0.0469 0 0 0.141 0 0.0675 0 0.0788 0.0566 0.077 0 0 0

Platelets 0.0573 0 0.0096 0.0107 0.027 0.0044 1e-04 0.0239 0.0677 0 0.0599 0 0.0623 0.0206 0 0.0265 0.0472 0 0.0392 0.0118

Preadipocytes 0.187 0.1466 0 0.0465 0.2628 0.2081 0.0663 0.0972 0.1636 0.0218 0.0041 0.0642 0.0454 0.0693 0.1082 0 0 0.1795 0.1402 0.0024

Sebocytes 0.0099 0.0221 0.0243 0.0234 0 0.0379 0.0178 0.0132 0 0.0238 0.0344 0.0261 0.0071 0.0118 0.0302 0.0248 0.0078 0.0315 0.0211 0.0175

Skeletal muscle 0.0011 0.0641 0.037 0.0256 0.0656 0.0142 0 0.0347 0.0358 0.0356 0.0011 0.0204 0.0532 0 0.0229 0.0132 0.053 0 0 0

Smooth muscle 0.0064 0 0.0145 0.0043 0 0 0 0 0 0.0087 0 0 0 0 0 0.0138 0.072 0 0.0585 0

Tgd cells 0 0.1873 0.1266 0.0328 0.0477 0.1053 0.299 0.269 0.0758 0.2189 0.2347 0.1882 0.2296 0.1802 0.2607 0.2235 0.1821 0 0.0287 0.129

Th1 cells 0 0.1483 0.2058 0.1199 0 0.0133 0.1318 0.0039 0.1812 0.1116 0.1389 0.1689 0.2998 0.0501 0.2063 0.1394 0.2497 0 0.0756 0.3414

Th2 cells 0 0.0632 0 0 0 0.0294 0.0418 0.0864 0 0.0584 0.0701 0.0824 0.0014 0.0718 0.0614 0.1132 0.0288 0 0 0.0065

Tregs 0.0919 0.0992 0.0987 0.0392 0.1134 0.0256 0.1141 0.0667 0.0722 0.0516 0.048 0.0083 0.0015 0.0657 0.0339 0 0.0181 0.1284 0.0704 0

aDC 0.0082 0.1759 0 0 0.223 0.0839 0.2522 0.2044 0.1015 0.1326 0.0941 0.0751 0 0.1524 0.1197 0.0762 0 0 0.0457 0

cDC 0.0062 0.0603 0 0 0.0869 0 0.1732 0.163 0.1764 0.1609 0.0887 0.0328 0 0.0901 0 0.0302 0 0.0795 0.0493 0

iDC 0.0587 0.0998 0 0.0362 0.2242 0.0019 0.0631 0.1373 0.221 0.0544 0.0996 0.0792 0 0.1697 0.0148 0.1004 0 0.1814 0.0745 0.0031

ly Endothelial cells 0.0218 0.0847 0.0212 0 0.3412 0.154 0 0.1038 0.102 0.0085 0 0 0.0176 0.0269 0 0 0.0215 0.0397 0.0072 0

mv Endothelial cells 0.1044 0.2274 0.0017 0 0.452 0.2602 0.12 0.197 0.168 0.1343 0.0469 0.0205 0.0894 0.1823 0.1098 0.0334 0 0.1301 0.1373 0.1054

naive B-cells 0 0 0 0 0 0 0 0.0106 0 0 0 0 0 0 0 0 0 0 0 0

pDC 0 0.0756 0 0 0.0761 0 0.1613 0.0787 0.0635 0.0369 0.0432 0.0546 0 0.0297 0.0803 0.0497 0 0 0 0

pro B-cells 0 0.0684 0.0456 0 0 0 0.0591 0.0859 0 0.0428 0.0787 0.0864 0.1419 0.0073 0.0679 0.0932 0.1008 0 0 0.088

ImmuneScore 0.1577 0.4121 0.2028 0.2284 0.7817 0.9439 1.1927 0.9149 0.6995 0.4277 0.3533 0.1859 0.2042 0.4685 0.6305 0.2556 0.2022 0.4176 0.4215 0.1412

StromaScore 0.3064 0.3164 0.0219 0.0266 0.5514 0.092 0.0225 0.3261 0.2253 0.0394 0.0762 0.1062 0.0575 0.0763 0.0315 0.0085 0.0707 0.2644 0.2106 0

MicroenvironmentScore 0.4642 0.7284 0.2247 0.255 1.3332 1.0359 1.2152 1.241 0.9248 0.4672 0.4295 0.2921 0.2617 0.5448 0.662 0.2641 0.273 0.682 0.632 0.1412

**Table 2 – xCell output for 150 bp sequencing samples**

S1 S2 S3 S4

Adipocytes 0.1768 0 0.1111 0.0187

Astrocytes 0.0214 0.0136 0 0.0262

B-cells 0 0 0.109 0.1794

Basophils 0 0.0147 0 0.0982

CD4+ T-cells 0 0.022 0.2402 0.4315

CD4+ Tcm 0 0 0.1388 0.1303

CD4+ Tem 0.076 0 0.388 0.4845

CD4+ memory T-cells 0.1032 0 0.2964 0.6017

CD4+ naive T-cells 0 0.0216 0.053 0.1742

CD8+ T-cells 0 0.0546 0 0.169

CD8+ Tcm 0 0.0604 0.0436 0.2372

CD8+ Tem 0.1599 0 0.1167 0.3894

CD8+ naive T-cells 0 0.0108 0.0638 0

CLP 0.0229 0.0385 0 0.0241

CMP 0.0151 0.0102 0 0

Chondrocytes 0.298 0.0968 0 0.0568

Class-switched memory B-cells 0 0.0269 0.1924 0.195

DC 0.1358 0 0.0077 0.3086

Endothelial cells 0.2595 0 0.0995 0

Eosinophils 0 0 0.2832 0

Epithelial cells 0 0.2244 0.165 0

Erythrocytes 0.021 0 0 0

Fibroblasts 0.3723 0 0 0

GMP 0.1909 0 0.1188 0.1187

HSC 0.2449 0 0 0

Hepatocytes 0 0 0.032 0

Keratinocytes 0 0.1738 0.114 0.0217

MEP 0 0 0 0

MPP 0.0583 0 0.1762 0.3246

MSC 0.3366 0.1364 0 0.1524

Macrophages 0.1486 0 0 0.2859

Macrophages M1 0.109 0 0.0179 0.1527

Macrophages M2 0 0 0 0.1267

Mast cells 0 0.009 0 0.0253

Megakaryocytes 0.0848 0.122 0.0905 0

Melanocytes 0.0048 0 0 0

Memory B-cells 0.0248 0 0.0387 0.0772

Mesangial cells 0.1018 0.1581 0 0.0706

Monocytes 0.0851 0 0.1386 0.0939

Myocytes 0.0713 0 0 0

NK cells 0.0195 0 0.0081 0.1558

NKT 0 0 0.0367 0.1168

Neurons 0.0253 0.0439 0.003 0

Neutrophils 0.0136 0 0.3847 0.0659

Osteoblast 0.0341 0.2271 0 0.144

Pericytes 0.0933 0 0 0

Plasma cells 0 0 0.1338 0.0374

Platelets 0 0.026 0.0202 0

Preadipocytes 0.0539 0 0.1245 0

Sebocytes 0 9.00E-04 0.0154 0

Skeletal muscle 0.0578 0 0 0

Smooth muscle 0 0.0714 0 0

Tgd cells 0.1614 0 0.0552 0.2542

Th1 cells 0.046 0 0 0.0471

Th2 cells 0.0801 0 0.0674 0.1076

Tregs 0.0064 0 0 0.0861

aDC 0.2334 0 0.0765 0.3

cDC 0.0189 0 0 0.2252

iDC 0.0639 0 0.0135 0.0655

ly Endothelial cells 0.1757 0 0.148 0

mv Endothelial cells 0.2659 0 0.2597 0.0993

naive B-cells 0 0 0 0.0232

pDC 0.1124 0 0.0213 0.2383

pro B-cells 0.0645 0 0 0.0684

ImmuneScore 0.2684 0.0571 0.781 1.1435

StromaScore 0.4043 0 0.1053 0.0094

MicroenvironmentScore 0.6727 0.0571 0.8863 1.1529
